# Identification of Tissue-Resident Natural Killer and T Lymphocytes with Anti-Tumor Properties in Ascites of Ovarian Cancer Patients

**DOI:** 10.3390/cancers15133362

**Published:** 2023-06-27

**Authors:** Elin Bernson, Oisín Huhn, Veronika Karlsson, Delia Hawkes, Maria Lycke, Valentina Cazzetta, Joanna Mikulak, James Hall, Anna M. Piskorz, Rosalba Portuesi, Domenico Vitobello, Barbara Fiamengo, Gabriele Siesto, Amir Horowitz, Hormas Ghadially, Domenico Mavilio, James D. Brenton, Karin Sundfeldt, Francesco Colucci

**Affiliations:** 1 Department of Obstetrics and Gynaecology, University of Cambridge School of Clinical Medicine, NIHR Cambridge Biomedical Research Centre, Addenbrooke’s Hosptial, Cambridge CB2 0QQ, UK; 2 Sahlgrenska Center for Cancer Research, Department of Obstetrics and Gynecology, Institute of Clinical Sciences, University of Gothenburg, 405 30 Gothenburg, Sweden; 3 Laboratory of Clinical and Experimental Immunology, IRCCS Humanitas Research Hospital, Via Manzoni 56, 20089 Rozzano, Italy; 4 Department of Medical Biotechnologies and Translational Medicine, University of Milan, 20122 Milan, Italy; 5 Cancer Research UK Cambridge Institute, University of Cambridge, Cambridge CB2 1TN, UK; 6 Unit of Gynecology, IRCCS Humanitas Research Hospital, Via Manzoni 56, 20089 Rozzano, Italy; 7 Unit of Pathological Anatomy, IRCCS Humanitas Research Hospital, Via Manzoni 56, 20089 Rozzano, Italy; 8 Department of Oncological Sciences, Lipschultz Precision Immunology Institute, Tisch Cancer Institute, Icahn School of Medicine at Mount Sinai, New York, NY 10029, USA; 9 AstraZeneca, Oncology R&D, Granta Park, Cambridge CB21 6GP, UK; 10 Department of Pathology, School of Medicine and Oral Health, Kamuzu University of Health Sciences, Mahatma Gandhi Road, Blantyre Private Bag 360, Malawi

**Keywords:** NK cells, ovarian cancer, NKG2A, tissue-resident lymphocytes

## Abstract

**Simple Summary:**

Ovarian cancer is the deadliest among gynecological cancers, and there is a huge demand for new treatments for these patients. Immunotherapy holds great potential in cancer treatment, but has not yet proven successful for the majority of ovarian cancer patients. To better understand the immunological landscape of the disease, we have characterized lymphocytes in patients with high-grade serous ovarian cancer. Natural killer cells and T cells are present in both primary tumors and in the metastasizing environment of ascites, a fluid in the abdominal cavity that is developed in many patients with ovarian cancer. Our data reveal that a large fraction of these natural killer cells and T cells express tissue-resident markers and the inhibitory receptor, NKG2A, and are able to kill ovarian cancer cells. In summary, we report a functional subset of lymphocytes that may be targeted in future immunotherapeutic approaches.

**Abstract:**

Women with ovarian cancer have limited therapy options, with immunotherapy being unsatisfactory for a large group of patients. Tumor cells spread from the ovary or the fallopian tube into the abdominal cavity, which is commonly accompanied with massive ascites production. The ascites represents a unique peritoneal liquid tumor microenvironment with the presence of both tumor and immune cells, including cytotoxic lymphocytes. We characterized lymphocytes in ascites from patients with high-grade serous ovarian cancer. Our data reveal the presence of NK and CD8^+^ T lymphocytes expressing CD103 and CD49a, which are markers of tissue residency. Moreover, these cells express high levels of the inhibitory NKG2A receptor, with the highest expression level detected on tissue-resident NK cells. Lymphocytes with these features were also present at the primary tumor site. Functional assays showed that tissue-resident NK cells in ascites are highly responsive towards ovarian tumor cells. Similar results were observed in an in vivo mouse model, in which tissue-resident NK and CD8^+^ T cells were detected in the peritoneal fluid upon tumor growth. Together, our data reveal the presence of highly functional lymphocyte populations that may be targeted to improve immunotherapy for patients with ovarian cancer.

## 1. Introduction

Ovarian cancer (OC) is characterized by the primary growth of tumor cells in the ovary or the fallopian tube. If untreated, it eventually spreads first to surrounding tissues and the abdominal cavity, and later, to distant organs. High-grade serous ovarian cancer (HGSC) is the most common subtype of OC, accounting for approximately 70% of diagnosed cases [1]. Due to the asymptomatic nature of this disease in the early stage, the majority of cases are diagnosed at later stages when the cancer has metastasized. Therefore, it has also been known as “the silent killer”. Despite there being high response rates to debulking surgery and chemotherapy, more than 80% of late-stage patients experience recurrent disease; thus, the overall survival is poor, with only 30% of patients surviving 5 years after diagnosis [2]. For the majority of HGSC patients with a recurrent disease, there is no curative treatment available, and there is a significant clinical need for novel treatment options. Immunotherapy has revolutionized cancer treatment for some patients with certain types of cancer; however, immunotherapy has yet to deliver satisfactory outcomes among patients with HGSC [3,4,5,6,7]. Understanding the immunological landscape of the disease is key to developing new immunotherapeutic approaches.

The spread of ovarian tumor cells into the abdominal cavity is often accompanied by the build up of peritoneal fluid or ascites. Within the ascites, both single tumor cells and tumor spheroids are present, together with immune cells, including natural killer (NK) cells and T cells. NK cells are innate lymphoid cells (ILCs) [8], which like CD8^+^ cytotoxic T cells, can kill tumor cells. However, while T cells require antigen-specific activation, NK cells can kill tumor cells without prior sensitization [9].

Immune cells in the tumor microenvironment may either halt tumor progression or facilitate it. One the one hand, NK and T cells, dendritic cells, macrophages and neutrophils contrast tumor progression with cell-mediated cytotoxicity, antigen presentation, pro-inflammatory cytokines and reactive oxygen species. On the other hand, regulatory T cells and myeloid-derived suppressor cells produce anti-inflammatory cytokines and angiogenesis-promoting factors that promote tumor progression [10]. Thus, the interplay between the different immune cells in the tumor microenvironment is important in immune surveillance and tumor progression. The role of NK cells in tumorigenesis and immune surveillance has been suggested based on studies reporting a higher cancer incidence among patients with impaired NK cell function or reduced NK cell cytotoxicity, as well as higher levels of tumor growth among mice with defective NK cells (reviewed in [5]).

The activation status of NK cells is determined by the balance of ligated activating and inhibitory receptors on their surface within the immunological synapse; a high rate of activation leads to the degranulation of cytotoxic granules into the target cells and/or the release of inflammatory cytokines. Among the inhibitory receptors, the dimer composed of CD94 and NKG2A (hereafter referred to as NKG2A) is expressed on about half of the peripheral blood NK cells and binds to the non-classical MHC class I molecule, HLA-E. Variable inhibitory killer-cell immunoglobin-like receptors (KIR) are expressed to various extents on subsets of NK cells and bind to classical MHC class I molecules HLA-A, -B or -C. NKG2A can regulate both adaptive and innate immunity as it is expressed not only on NK cells, but also on conventional CD8^+^ T lymphocytes [11], as well as unconventional T cell populations, such as NKT cells [12] and γδ T cells [13,14].

Under certain conditions, NK and T cells also express other inhibitory receptors, such as PD-1, CTLA-4, TIM-3, LAG-3 and TIGIT [15,16,17]. These inhibitory receptors, when engaged with their cognate ligand in the effector phase of an immune response, raise the activation threshold and suppress NK cell activation, as well as T cell activation. Therefore, targeting these receptors with monoclonal antibodies may be a potential immunotherapy treatment for certain types of cancer, an approach known as an “immune checkpoint blockade”. However, an immune checkpoint blockade directed towards NK cells has yet not been shown successful in larger clinical trials [18,19]. Moreover, the checkpoint blockade of PD-1 or CTLA-4 has only generated modest results in OC, with a few patients responding [4,7]. There are other immunotherapeutic approaches under investigation. One is the adoptive cell transfer of autologous or allogeneic effector cells (including in vitro expanded tumor-specific T cells from tumor infiltrating lymphocytes; TILs, engineered T or NK cells expressing a chimeric antigen receptor (CAR) construct, or in vitro activated NK cells). Another one is immune cell activation using cytokines to stimulate lymphocyte cytotoxicity and cytokine production [4,10,20]. NK cell transfer, in contrast to T cell transfer, is advantageous because it does not require MHC matching nor antigen specificity. While the transfer of allogeneic NK cells has been reported as feasible [21], more research is ongoing, for instance, to identify optimal sources of NK cells [22,23,24].

In contrast to other ILCs, which are found almost exclusively in tissues, NK cells are present both in the blood, where they represent 2–15% of circulating lymphocytes, and in tissues. In the blood, NK cells are commonly divided into CD56^dim^ and CD56^bright^ NK cells. CD56^dim^ NK cells are more abundant and have a higher cytotoxic potential, whereas CD56^bright^ NK cells are less common, less cytotoxic and show a higher cytokine production capacity [25]. By contrast, CD56^bright^ are more abundant in both healthy and neoplastic tissues [26,27]. Previous studies have reported that CD56^bright^ cells are also more abundant in OC ascites than they are in peripheral blood [28,29,30], and just as their peripheral blood CD56^bright^ NK cell counterpart, these CD56^bright^ NK cells may possess a lower cytotoxic potential. However, recent research from our lab and others’ labs show heterogeneity among tissue-resident NK cells with different subsets and functions [31,32,33]. Here, using high-dimensional mass cytometry, we characterized the NK cells present in the ascites of women with HGSC to analyze cell composition and function beyond the CD56^bright^ and CD56^dim^ dichotomy. Our data reveals that the majority of NK cells in the malignant environment express tissue-residency markers, with a high expression level of the inhibitory receptor, NKG2A. Moreover, the functional characterization of this subset shows that they respond towards ovarian tumor cells.

## 2. Materials and Methods

### 2.1. Patients and Samples

Ascites and matched peripheral blood samples from 8 patients diagnosed with HGSC (stage 2C-4B) were included in this study. The inclusion criteria comprised chemo naïve patients with confirmed high-grade serous ovarian cancer (HGSC). Matched ascites and blood samples were received from the Sahlgrenska University Hospital (Gothenburg, Sweden) or the Cambridge University Hospitals NHS Foundation Trust (Cambridge, UK) after written consent was obtained from the patients. Samples were collected during debulking surgery or paracentesis prior to surgery. The study was approved by the regional ethics board in Gothenburg (201-15) or by the institutional review board in Cambridge (08/H0306/61). The collection of matched HGSC tumor and blood samples from 5 patients was performed at the Humanitas Research Hospital (HRH) after it was ethically approved by the Institutional Review Board (IRB) of HRH (606/22). All enrolled patients were enrolled according to the Declaration of Helsinki and signed a written informed consent form. Details of the patient samples can be found in Table A1.

After collection, ascites samples were filtrated in two steps using a muslin/180 µm nylon net filter, which was followed by 40 µm nylon net filter (Merck Millipore, Darmstadt, Germany) to achieve a single cell suspension. For NK isolation, dextran sedimentation was used to remove the erythrocytes from peripheral blood and filtrated ascites, followed by gradient centrifugation with lymphoprep (Stemcell Technologies UK Ltd., Cambridge, UK) to obtain a mononuclear cell suspension. Cells were washed in RPMI 1640. NK cells were isolated using a negative NK isolation kit (Miltenyi Biotech, Bergisch Gladbach, Germany) according to the protocol provided by the manufacturer. Cells were suspended in Gibco Recovery Cell Culture Freezing Medium (Gibco, Thermofisher, Waltham, MA, USA) or fetal bovine serum supplemented with 10% of dimethylsulfoxide (Sigma-Aldrich, St. Louis, MO, USA) and cryopreserved in liquid nitrogen for further analysis.

A freshly isolated primary tumor tissue sample from HGSC patients was dissociated via enzymatic digestion in a gentleMACS™ Dissociator (Miltenyi Biotech) with 2 mg/mL of collagenase D (Sigma-Aldrich) and 100 µg/mL of hyaluronidase (Sigma-Aldrich) for 45 min at 37 °C/5% CO_2_. Cells were then filtered through a 100 µm cell strainer (Corning, New York, NY, USA) and washed in Hank’s Balanced salt solution buffer without Ca^2+^ and Mg^2+^ (HBSS−/−; Euroclone SpA, Pero, Italy). Cells were filtered through a 70 µm cell strainer (Corning) and washed in HBSS−/−. At the end, the single-cell suspension was frozen in fetal bovine serum (FBS; Lonza, Basel, Switzerland) supplemented with 10% of dimethylsulfoxide (DMSO; Lonza) in liquid nitrogen for further analysis. The peripheral blood mononuclear cells (PBMCs) were isolated from freshly isolated blood of HGSC patients using a Lympholyte^®^ Cell Separation density gradient solution (Cederlane Laboratories: Burlington, ON, Canada) following the manufacturer’s instructions.

For the initial discovery step of CyTOF analysis, we used 3 peripheral blood and ascites samples collected at the Cambridge site. For the validation step and the functional assays, we used 5 peripheral blood and ascites samples collected at the Gothenburg site. A further validation step was conducted using 5 peripheral blood and primary tumor biopsy samples collected at the Milan site. Ten peripheral blood and ascites/primary tumor samples collected at the Gothenburg and Milan sites were shipped on dry ice to Cambridge. All phenotyping and functional assays were performed at the University of Cambridge.

### 2.2. Mice

Female C57BL/6 mice were obtained from Charles River (Wilmington, MA, USA) and hosted according to UK Home Office guidelines. All experiments were approved by the University of Cambridge Ethical Review Panel and carried out in accordance with Home Office Project License PPL 2363781.

For the mouse OC model, 5 × 10^6^ ID8 *Trp53*^−/−^ cells [34], kindly provided by Prof Iain McNeish (Imperial College London, UK), were intraperitoneally injected into C57BL/6 mice. The endpoint was defined as 6 weeks post-tumor injections, or earlier if the mice demonstrated clinical signs of ill health.

Mice were killed via cervical dislocation, after which peritoneal fluid and spleens were collected. Spleens were minced and passed through a 70 µm cell strainer, after which red blood cells were lysed using BD Pharm Lyse buffer (BD Biosciences, San Diego, CA, USA). Peritoneal fluid was washed several times in RPMI 1640. In cases of red blood cell contamination, samples were treated with BD Pharm Lyse buffer (BD Biosciences). Single-cell suspensions were washed in RPMI 1640 and used for downstream analysis and assays. For functional assays, NK cells were isolated from splenocytes using negative NK isolation kit (Miltenyi Biotech) according to the manufacturer’s protocol.

### 2.3. Cell Lines

Human ovarian cancer cell line, OVCAR-3, and myelogenous leukemia cell line, K562, were both provided by the Department of Infectious Diseases at the University of Gothenburg. OVCAR-3 cells were cultured in RPMI 1640 (Gibco, Thermofisher, Waltham, MA, USA) supplemented with 20% heat-inactivated fetal bovine serum (FBS), 1% L-Glutamine (Gibco), 1% Pen Strep (Gibco) and 0.01 mg/mL bovine insulin (Sigma-Aldrich) in a humidified incubator with 5% carbon dioxide at 37 °C. Cells were passaged before reaching confluency and detached using 0.5 mM EDTA (Gibco) in PBS followed by cell scraping. K562 cells were cultured in Iscove’s Modified Dulbecco’s Medium (Gibco) supplemented with 10% FBS, 1% Pen Strep, 1% Sodium Pyruvate (Gibco) and 1% L-glutamine. The HLA-E-transfected TAP-deficient T2 lymphoblast cell line, kindly provided by Dr Christina Bade-Döding (Hannover Medical School, Germany), was maintained in RPMI 1640, 10% FBS and 1% Pen Strep. The mouse ovarian surface epithelial cell line, ID8 *Trp53*^−/−^ [34], was maintained in DMEM (Sigma-Aldrich) supplemented with 4% FBS, 5 µg/mL Insulin, 5 µg/mL Transferrin 5 ng/mL Sodium selenite and 1% Pen Strep.

### 2.4. Functional Assays

NK cells isolated from the ascites of HGSC patients were thawed and stimulated over night with IL-2 (500 IU/mL, Peprotech, Cranbury, NJ, USA). Cells were washed and co-incubated with target cells (OVCAR-3, K562 or T2E) at a 1:1 ratio for 4 h. CD107a mAb (BUV395; BD Horizon, San Diego, CA, USA) was added at the beginning of the assay, and protein transport inhibitor cocktail (eBioscience, San Diego, CA, USA) was added after 1 h.

Mouse NK cells were co-incubated with ID8 *Trp53*^−/−^ cells at a 1:1 ratio for 4 h in the presence of CD107a (PE; Biolegend, San Diego, CA, USA). As a positive control, PMA/Ionomycin cocktail (eBioscence) was added to NK cells. In specified experiments, ID8 *Trp53*^−/−^ cells were stimulated with interferon-γ (IFN-γ, 10 ng/mL; Peprotech) for 48 h at 37 °C with 5% CO_2_ prior to the degranulation assay. The degranulation of specified NK subsets was analyzed via flow cytometry.

### 2.5. Phenotyping

If not specified otherwise, cells were stained for functional and phenotypical markers and analyzed via flow cytometry using an LSR Fortessa II (BD). When specified, cells were analyzed using mass cytometry (CyTOF) with a Helios mass cytometer (Fluidigm, South San Francisco, CA, USA). When the antibodies required conjugation, MaxPar X8 labeling kits (Fluidigm) were used according to the manufacturer’s protocol. Cells were stained with viability marker, LIVE/DEAD Fixable Near-IR (Invitrogen, Waltham, MA, USA) or Fixable Viability Dye eFluor 780 (Thermofisher, Waltham, MA, USA), which was followed by Fc-receptor blocking with TruStain fcX (Biolegend) for mouse experiments and extracellular staining. For intracellular staining, cells were treated with the eBioscience Fixation/Permeabilization kit (Thermofisher). To obtain the HLA-E expression of target cell lines, target cells from culture were stained with viability marker LIVE/DEAD Fixable Near-IR (Invitrogen), followed by staining for HLA-E expression. Detailed information about the antibodies used can be found in Table A2 and Table A3 for mass and flow cytometry, respectively. Data were analyzed using FlowJo (BD Biosciences; v.10 or later).

### 2.6. Statistical Analysis

Multiple group comparisons were analyzed using one-way ANOVA. For pairwise comparison, Student’s *t*-test was used. A *p*-value < 0.05 was considered to be significant. tSNE analysis were performed using the R package cytofkit from Bioconductor [35]. Markers included in each analysis are highlighted in Table A2.

## 3. Results

### 3.1. Ovarian Cancer Ascites Conatins a Large Subset of Tissue-Resident NK Cells

**Figure 1:**
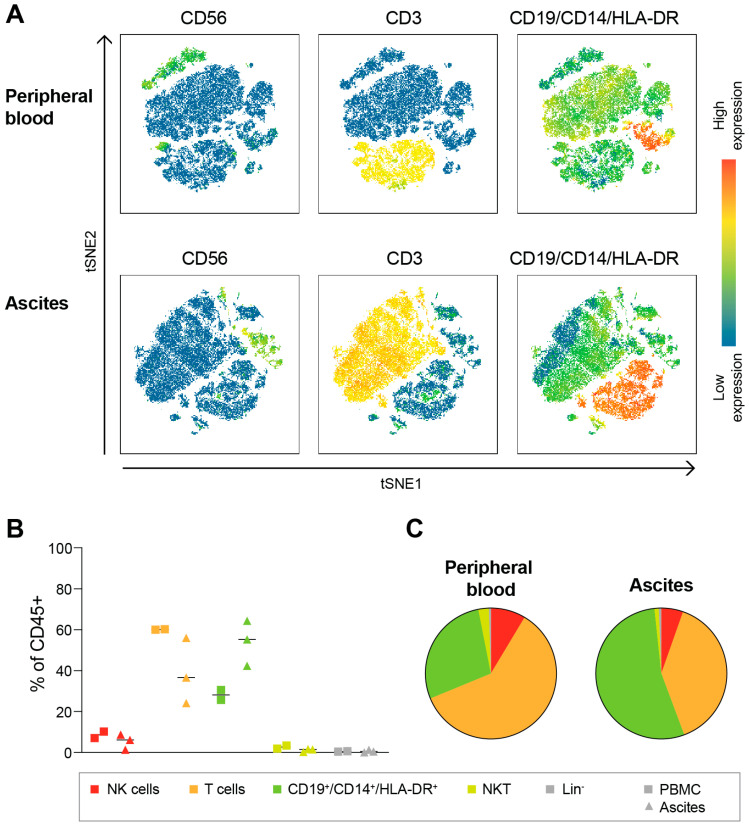
Total immune cell-type composition in HGSC ascites. (**A**) tSNE landscape of CD45^+^ cells in peripheral blood and ascites from one representative OC patient. Red represents high expression level of the noted marker, while blue represents low expression level. (**B**,**C**) Frequencies of specified immune cell subsets in peripheral blood and OC ascites for individual patients (**B**) or mean of all patients (**C**). Lin^−^ defined as cells negative for CD56, CD3, CD19, CD14 and HLA-DR). n = 3 ascites; n = 2 blood.

In the discovery step of the study, we used three peripheral blood and ascites samples collected at the Cambridge site. Cells from fresh HGSC ascites and matched peripheral blood were stained using a CyTOF panel including 40 markers (see Table A2). The immune composition of CD45^+^ cells was visualized in a tSNE landscape, as shown in Figure 1, where CD56^+^CD3^−^ NK cells were separate from CD3^+^ lymphocytes in both peripheral blood and ascites samples. CD19, CD14 and HLA-DR were included in the same channel, and thus, appear together in the plot. Based on the tSNE landscape, cell subsets were manually gated into CD45^+^CD14^−^CD19^−^HLA-DR^−^CD3^−^CD56^+^ NK cells, CD45^+^CD14^−^CD19^−^HLA-DR^−^CD3^+^CD56^−^ T cells, CD45^+^CD14^−^CD19^−^HLA-DR^−^CD3^+^CD56^+^ NKT cells and CD45^+^CD14^+^CD19^+^HLA-DR^+^CD3^−^CD56^−^ B cells/monocytes. As demonstrated in Figure 1, T cells account for roughly 40% of all CD45^+^ cells in the ascites and 60% in the peripheral blood, while NK cells represent a mean value of 6 or 8% of the CD45^+^ cell compartment in ascites and peripheral blood, respectively. Unconventional NKT cells represent around 1% of all immune cells.

Previous studies on NK cells in ascites (aNK) from OC patients have commonly grouped NK cells into conventional CD56^bright^ and CD56^dim^ NK cells following the definition of peripheral blood NK (pbNK) cells. However, based on our previous work with the heterogeneity of decidual NK cells [31], we hypothesized that aNK cells may present with unique NK subpopulations. Therefore, we used high-dimensional cytometry, including 34 NK cell markers, to further assess the aNK subsets. In addition to conventional pbNK-like subsets, we could identify subsets of aNK cells that expressed combinations of the tissue-associated integrins, CD49a [α1(CD49a)β1] and/or CD103 [αE(CD103)β7], which are important for the retention of tissue resident lymphocytes [27]. These aNK cells which are positive for at least CD49a or CD103, we hereafter referred to as tissue-resident ascites NK (traNK) cells (Supplementary Figure S1).

**Figure 2:**
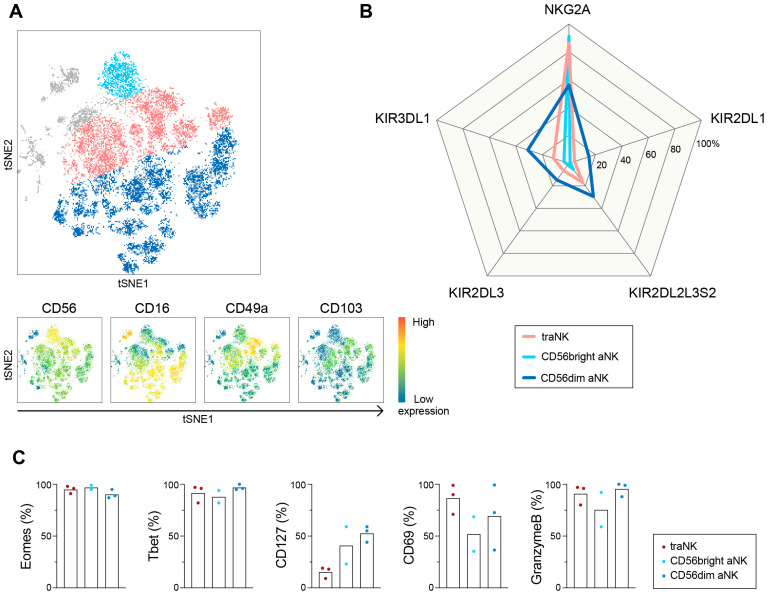
A large subset of tissue-resident NK cells within the OC ascites compartment. (**A**) tSNE landscape of Live CD45^+^CD14^−^CD19^−^HLA-DR^−^CD3^−^CD56^+^ cells in OC ascites. Three subsets of aNK cells are overlaid: tissue-resident aNK (traNK) cells expressing CD49a and/or CD103 (pink), and two subsets negative for tissue-resident markers and with dim CD56 expression and high CD16 expression level (dark blue), or bright CD56 expression (light blue). Lower graphs show expression intensity of specified markers, with red being the highest expression level and blue the lowest expression level. (**B**) Spider plot shows frequencies of specified inhibitory markers on aNK cell subsets. (**C**) Frequencies of specified markers on aNK cell subsets. n = 3.

We then performed unsupervised tSNE analysis on Live CD45^+^CD14^−^CD19^−^HLA-DR^−^CD3^−^CD56^+^ NK cells and overlaid the CD56^bright^ aNK, CD56^dim^ aNK and traNK cells as identified via 2D gating (Supplementary Figure S1). Three aNK subsets were separated out on the generated tSNE landscape (Figure 2A), and we went on to characterize the expression of a number of NK-related markers. Our results confirm that CD56^bright^ and CD56^dim^ aNK cells resemble conventional pbNK in that the expression of KIRs and CD57 is restricted to the CD56^dim^ subset, while the majority of CD56^bright^ aNK cells are positive for NKG2A (Figure 2B and Figure S2). traNK cells are also highly positive for NKG2A, while their KIR expression level is lower than it is in CD56^dim^ aNK cells (Figure 2B). TraNK cells were further characterized as uniformly positive for NKp30, NKp46, NKG2D and CD7, which makes them similar to CD56^bright^ aNK cells, and with MFI values of the activating receptors NKp30, NKp46 and NKG2D, which is similar to those of CD56^bright^ aNK cells. Moreover, all traNK cells are positive for CD161, defining a pro-inflammatory function of cells [36], while the expression levels of CD57 and DNAM-1 are low. We also noticed a high frequency of CD9^+^ aNK cells in all subsets, which makes aNK cells resemble decidual NK cells, as earlier described in [37] (Supplementary Figure S2).

Transcription factors, Eomes and Tbet, are expressed by conventional NK cells, but only some specific ILC subsets express both [8]. For example, Eomes^+^T-bet^+^ CD127^low^ intra-epithelial (ie) ILC1-like cells have been described in the tumor microenvironment of head and neck squamous cell cancer [38,39]. Our data revealed that all subsets of HGSC aNK cells express both Eomes and Tbet (Figure 2C), and similarly to the ieILC1-like cells described in head and neck cancer, traNK also expresses low levels of CD127 as compared to those of CD56^bright^ and CD56^dim^ aNK cells. Similar to earlier reports [28,40,41] we found that CD69 is expressed at a high frequency in the traNK subset, confirming its tissue-resident state or suggesting activation. Importantly, we noticed that Granzyme B is expressed in a high proportion of both aNK cells, thus suggesting a general cytolytic potential in traNK, CD56^bright^ and CD56^dim^ aNK cells (Figure 2C).

### 3.2. All Subsets of aNK Cells Are Highly NKG2A^+^

**Figure 3:**
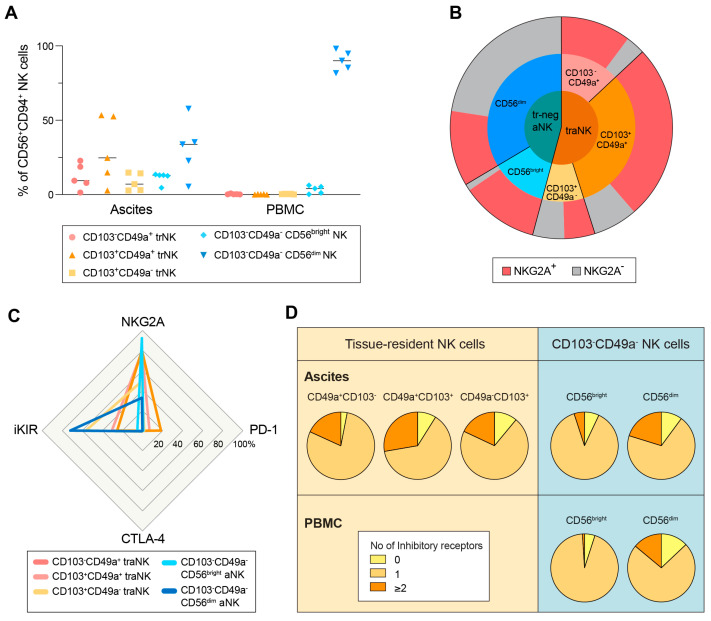
High expression level of NKG2A in the traNK cell subset. (**A**) NK cell subset distribution in ascites samples, with patient-matched PBMC. (**B**) Pie chart shows mean aNK subset distribution of (**A**) in tissue-resident (trNK) and CD49a^−^CD103^−^ (tr-neg NK) subsets. (**C**) Spider plot shows mean expression of specified inhibitory receptors within each aNK subset. (**D**) Pie charts show co-expression of inhibitory receptors (NKG2A, iKIR, PD-1 and/or CTLA-4) on NK subsets in ascites samples (aNK) and matched PBMCs (pbNK). n = 5.

In the validation step of our study, we used five peripheral blood and ascites samples collected at the Gothenburg site. Since a functional dichotomy was found in the tissue-resident CD49a^−^CD103^+^ and CD49a^+^CD103^+^ T cells [42], before evaluating the function of traNK cells, we stratified them into three subgroups: CD49a^+^CD103^−^, CD49a^+^CD103^+^, and CD49a^−^CD103^+^ (Figure 3A,B; gating strategy in Supplementary Figure S3). The vast majority of the CD49a^+^ traNK cells are NKG2A^+^ (Figure 3B). In contrast to NKG2A expression, the expression of other inhibitory molecules including PD-1, CTLA-4 and inhibitory KIRs is not as pronounced, and the co-expression of two or more inhibitory receptors are in a similar range in traNK cells and CD56^dim^ conventional aNK and pbNK cells (Figure 3C,D, Supplementary Figure S3).

### 3.3. NKG2A^+^ trNK Cells Are Present also in the Primary Tumor Microenvironment in HGSC

**Figure 4:**
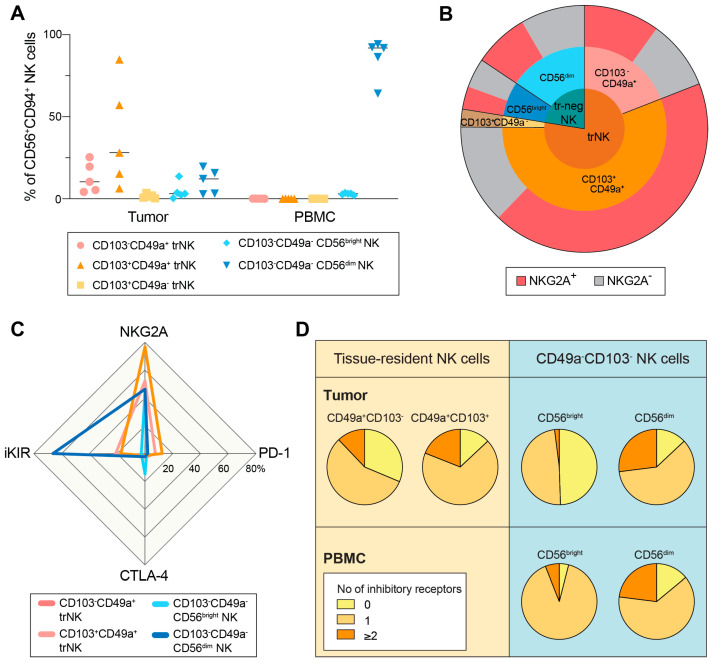
NKG2A^+^ trNK cells are present in the primary tumor environment of HGSC. (**A**) NK cell subset distribution in tumor samples, and with patient-matched PBMC. (**B**) Pie chart shows mean NK subset distribution of (**A**) in tissue-resident (trNK) and CD49a^−^CD103^−^ (tr-neg NK) subsets. Due to low numbers of CD49a^−^CD103^+^ trNK cells, the frequency of NKG2A expression could not be shown. (**C**) Spider plot shows mean expression of inhibitory receptors within each aNK subset. (**D**) Pie charts show co-expression of inhibitory receptors (NKG2A, iKIR, PD-1 and/or CTLA-4) on NK subsets in tumor samples and matched PBMCs. n = 5.

In the second part of our validation step, and to test the hypothesis generated during discovery and validation, we used five peripheral blood and primary tumor samples collected at the Milan site. We phenotypically characterized NK cells from the primary tumor of five patients diagnosed with HGSC. Similar to the ascites compartment, there is a large fraction of NKG2A^+^ trNK cells in the primary tumor (Figure 4A,B). In contrast to the ascites compartment, the trNK subset in the primary tumor environment is almost completely made up of CD49a^+^. On the other hand, and like traNK cells, small fractions of trNK cells in the primary tumor environment express fewer iKIRs, PD-1 and CTLA-4 cells, with the co-expression of >2 inhibitory receptors in less that 20% of the subset (Figure 4C,D). Therefore, NKG2A is the predominant inhibitory receptor expressed in tissue-resident NK cells in both primary tumors and ascites.

### 3.4. traNK Cells Respond to Ovarian Tumor Cells

**Figure 5:**
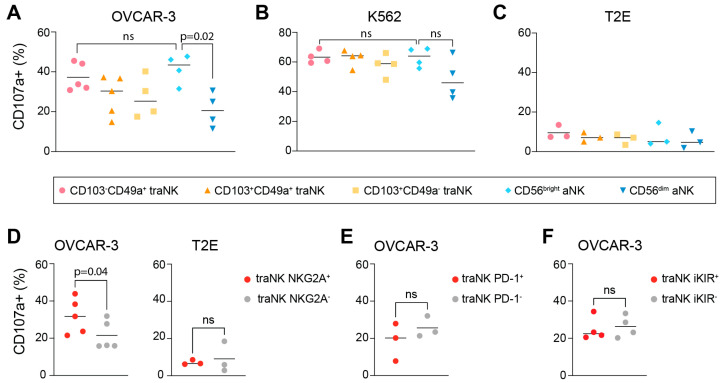
NKG2A+ traNK respond towards OC cells by degranulation. (**A**–**C**) Degranulation of specified aNK cell subsets towards OVCAR3 cells (**A**), K562 cells (**B**) and T2E cells (**C**). n = 5. ANOVA followed by Sidak’s multiple comparison test. (**D**–**F**) Degranulation towards specified cell lines (OVCAR-3 or T2E cells) within traNK cells that are positive or negative for NKG2A (**D**), PD-1 (**E**) or iKIRs (**F**), respectively. n = 5; paired-test, ns: not significant.

To test the functionality of aNK subsets, NK cells isolated from HGSC ascites were co-cultured with the human OC cell line OVCAR-3 or the standard NK target erythroleukaemia cell line, K562; the latter one lacked the expression of HLA-E or other HLA molecules. As shown in Figure 5A, CD49a^+^CD103^−^ traNK and CD56^bright^ aNK showed the highest degranulation levels (mean 37% and 43%, respectively), while CD49a^−^CD103^+^ traNK cells and CD56^dim^ aNK have a lower degranulation capacity (25% and 21%, respectively). Intriguingly, CD49a^+^CD103^−^ traNK and CD56^bright^ aNK marked the aNK populations with the highest fraction of NKG2A^+^ cells. Thus, we compared the degranulation responses of NKG2A^+^ vs. NKG2A^−^ populations of traNK cells and could conclude that the NKG2A^+^ population degranulated more towards the OC cell line, OVCAR3 (Figure 5D). In contrast, PD-1^+^ or iKIR^+^ traNK cells did not degranulate more than their PD-1^−^ or iKIR^−^ counterparts did (Figure 5E,F). Degranulation towards the HLA-E- K562 cells did not differ between any of the aNK subsets (Figure 5B). Thus, the expression of NKG2A, but not of other inhibitory receptors, correlates with greater response to ovarian tumor cells.

As NKG2A^+^ cells are inhibited by HLA-E-expressing target cells, we investigated the HLA-E expression of the OVCAR3 cell line and found that the cells expressed low levels of HLA-E [43]. In order to investigate the degranulation capacity of aNK cell subsets against HLA-E expressing target cells, we used the TAP-deficient T2 lymphoblast cell line transfected with an HLA-E construct that only expresses HLA-E when it is provided with suitable peptides (T2E) [44]. Accordingly, the aNK degranulation level towards HLA-E-expressing T2E cells was markedly lower compared to the degranulation level towards K562 or OVCAR3 cells (Figure 5C). Moreover, both NKG2A^+^ and NKG2A^−^ traNK cells responded similarly and with a low level of degranulation of HLA-E-expressing T2E target cells (Figure 5D), suggesting that NKG2A on traNK cells marks the functional potential suppressed via the expression of HLA-E on target cells.

### 3.5. Presence of Tissue-Resident CD8^+^ T Cells in Ascites and Tumor Environment of HGSC

**Figure 6:**
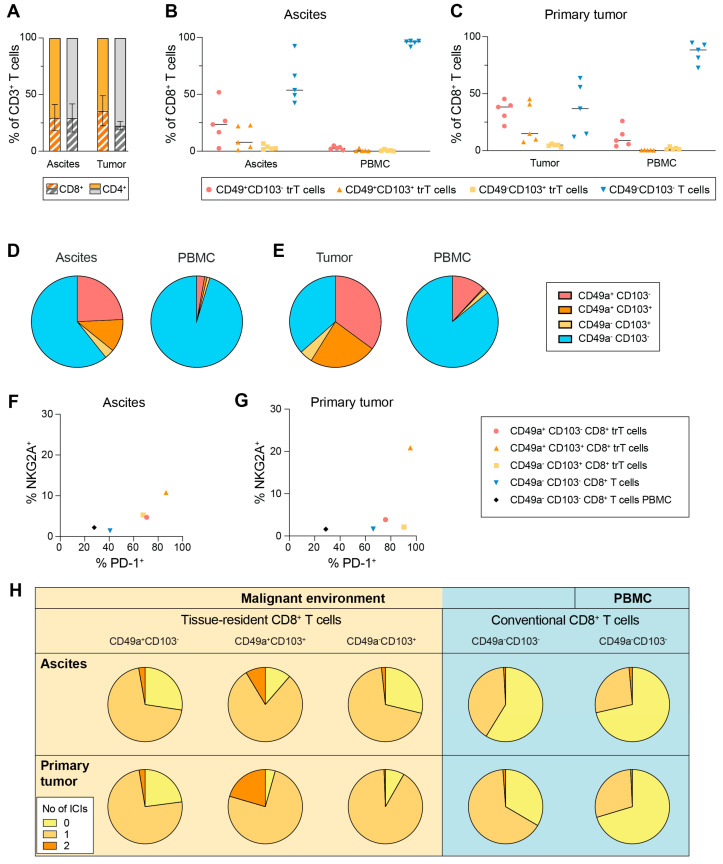
Presence of tissue-resident CD8^+^ T cells in ascites and tumor environment of OC. (**A**) Distribution of CD8^+^ and CD4^+^ T cells in ascites and primary tumor (orange), and in patient-matched PBMC samples (grey). (**B**,**C**) Distribution of CD8^+^ tissue-resident and conventional T cells in ascites (**B**) and primary tumor tissue (**C**), with patient-matched PBMC samples. (**D**,**E**) Mean distribution of specified CD8^+^ T cell subsets in ascites (**D**) and tumors (**E**), with patient-matched PBMC samples. (**F**,**G**) Mean frequency of NKG2A^+^ and PD-1^+^ CD8^+^ T cell subsets in ascites (**F**) and primary tumor tissue (**G**), with patient-matched PBMC samples. (**H**) Co-expression of inhibitory receptors on CD8^+^ T cell subsets in ascites, primary tumor tissue and matched PBMC. n = 5.

Our initial analysis showed that the most common immune cells in ascites from OC patients are T cells. Because T cells can also express NKG2A [45,46,47], we asked whether ascites and/or primary tumor T cells express high levels of NKG2A. Samples from both ascites and primary tumor from patients with HGSC were used for phenotyping. Mean values of 30% and 36% of all T cells were CD8^+^ in the ascites and tumors, respectively (Figure 6A). Within the CD8^+^ T cell population, 39% and 63% in ascites and tumors, respectively, expressed at least one of the tissue-resident markers CD49a and CD103 (Figure 6B–E; hereafter denoted as trCD8^+^ T cells). Next, we investigated the NKG2A expression in the different T cell subsets. We noted an increased expression level of NKG2A in the tissue-resident subsets as compared to that of conventional CD8^+^ T cells in the malignant environment, or CD8^+^ T cells in peripheral blood (mean values shown in Figure 6F,G and individual values in Supplementary Figure S4). As expected, a high frequency of CD8^+^ T cells are PD-1^+^, with the highest expression level in the tissue-resident subsets. The co-expression of PD-1 and NKG2A is low in all subsets except in the CD49a^+^CD103^+^ trCD8^+^ T cell subset, with a mean of 9% and 20% of them being double-positive for both inhibitory receptors in ascites and tumors, respectively (Figure 6H). For CD4^+^ T cells subsets in OC ascites and the tumor microenvironment, see Supplementary Figure S5.

### 3.6. In Vivo Phenotype of Mouse NK and T Cells in Ascites

**Figure 7:**
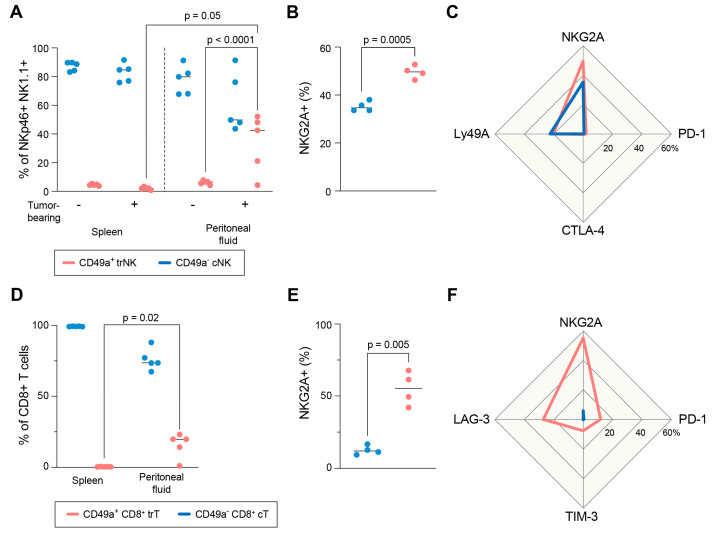
Tissue-resident NK and CD8^+^ T cells are present in peritoneal fluid upon tumor growth. (**A**) Frequency of trNK and cNK in spleen and peritoneal fluid with no tumor or 6 weeks after ID8 tumor injection. (**B**) Frequency of NKG2A^+^ trNK and cNK cells in peritoneal fluid 6 weeks after ID8 tumor injection. (**C**) Expression of inhibitory receptors in trNK and cNK cell subsets in peritoneal fluid 6 weeks after ID8 tumor injection. (**D**) Frequency of CD8^+^ trT and cT cells in spleen and peritoneal fluid 6 weeks after ID8 tumor injection. (**E**) Frequency of NKG2A^+^ CD8^+^ trT and cT cells in peritoneal fluid 6 weeks after ID8 tumor injection. (**F**) Expression of inhibitory receptors in CD8^+^ trT and cT cell subsets in peritoneal fluid 6 weeks after ID8 tumor injection. One-way ANOVA (**A**,**D**) or paired t-test (**B**,**E**).

In order to investigate the effect of NKG2A inhibition in an in vivo setting, we used a model of mouse OC cells based on the intraperitoneal (i.p.) administration of the ID8 mouse ovarian surface epithelial cell line (derived from C57BL/6 mice) into syngeneic wild-type C57BL/6 mice. Upon administration, ID8 cells establish tumors and induce ascites production [48]. To assess the immune compartment in the tumor microenvironment in peritoneal fluid, the ascites samples were analyzed using flow cytometry. Like OC-associated ascites in humans, we could detect a subset of traNK cells in mice ascites 6 weeks after tumor injection (Figure 7A). The frequency of traNK cells in the ascites of tumor-bearing mice was significantly higher the trNK cells in the peritoneal fluid of healthy control mice, suggesting the disease-driven infiltration/expansion of traNK cells. Similarly, we detected an increased frequency of CD8^+^ trT cells after tumor injection compared to that in the spleen (Figure 7D). Like for human cells, both tissue-resident mouse populations express NKG2A to a higher extent than the cell subsets resembling their conventional counterparts do (Figure 7B,E), while the increase in other inhibitory receptors are not as pronounced (Figure 7C,F).

**Figure 8:**
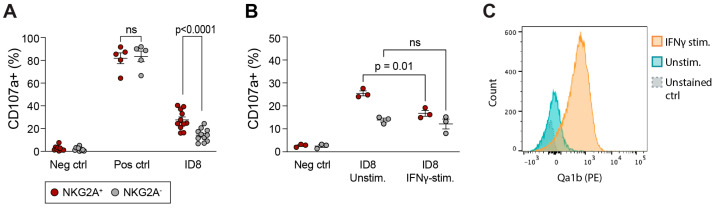
NKG2A expression on mouse NK cells correlates with higher degranulation towards ID8 cells. (**A**) Degranulation in NKG2A^+^ and NKG2A^−^ splenic mouse NK cells towards ID8 target cells. In positive control, NK cells were stimulated with PMA/Ionomycin. (**B**) Degranulation in NKG2A^+^ and NKG2A^−^ splenic mouse NK cells towards ID8 target cells that were previously stimulated with IFNγ. (**C**) Representative staining of Qa-1^b^ expression on ID8 cells that were either IFNγ-stimulated or unstimulated. One-way ANOVA followed by Sidak’s multiple comparison test, ns: not significant.

To investigate NK cell reactivity towards the ID8 cell line in vitro, we co-incubated splenic NK cells with ID8 cells for 4 h, after which CD107a expression was evaluated. While no difference in degranulation was seen between NKG2A^+^ and NKG2A^−^ NK cells in the positive control condition (response to PMA/Ionomycin), significantly more NKG2A^+^ NK cells degranulated in response to ID8 cells (Figure 8A). The murine NKG2A ligand in C57BL/6 mice is the non-classical MHC molecule, Qa-1^b^. To test the impact of NKG2A ligand expression on the degranulation of NKG2A^+^ NK cells, we increased the Qa-1^b^ expression level in ID8 cells by culturing them with IFNγ for 48 h prior to co-culturing them with NK cells (Figure 8C). While NKG2A^−^ NK cells were not affected by the increased expression of the NKG2A ligand, Qa-1^b^, the NKG2A^+^ subset degranulated to a significantly lower extent when they were exposed to ID8 cells with a high Qa-1^b^ expression level (Figure 8B). Therefore, in mouse NK cells, like in human NK cells, the expression of NKG2A marks a functional potential that is suppressed by the cognate ligand on tumor cells.

## 4. Discussion

How NK cells in the ascites of OC patients may affect the course of the disease is unclear. While high numbers of CD16^+^ NK cells in ascites or pleural effusions in OC correlates with a poor overall survival [49], other studies have suggested the opposite [30]. The discrepancy in the outcomes may partly be explained by the fact that NK cells have been defined based on different markers; some studies included only CD16^+^ cells in the NK cell group, while others also included CD56^bright^ CD16^−^ NK cells. Both these previous studies, however, do not consider further subpopulations of NK cells. In this study, we set out to characterize the NK cell subsets present in OC ascites using high-dimensional cytometry. Our data reveals that a large fraction of the NK cells present in OC ascites express tissue-residency markers. Moreover, the expression level of the transcription factors, EOMES and T-bet, is high in the tissue-resident population, while the expression level of CD127 is decreased as compared to that of conventional NK cells.

This large fraction of tissue-resident NK cells in the ascites is similar to that of ieILC1 cells described in lung, colorectal and head and neck tumors [38,39,50], which have cytolytic potential [38,50], particularly CD49a^+^ ieILCs in HNSCC [38]. This is consistent with our data showing that the highest response rate to the OC cell line OVCAR-3 was detected in the CD49a^+^ traNK subset. This subset also had the highest frequency of NKG2A expression. Moreover, when comparing the NKG2A^+^ vs. NKG2A^−^ traNK responses it was significantly higher in the NKG2A^+^ subset, while the difference was reduced in response to high levels of HLA-E-expressing target cells. However, as the NKG2A–HLA-E axis was not specifically blocked, we cannot prove that the difference is solely dependent on the high HLA-E expression level. Within the ascites NK cells that did not express CD49a and CD103, surprisingly, the CD56^bright^ subpopulation degranulated significantly more than the CD56^dim^ subpopulation did. This may seem to be counterintuitive, as peripheral blood CD56^dim^ NK cells are known as the more cytotoxic subset [25]. However, as pointed out above, peripheral blood and tissue NK cells do have different properties.

Beyond OC ascites, we identified similar tissue-resident NK cell subsets in primary OC tumors, suggesting that these subsets are not specific to the ascitic microenvironment. Moreover, a high fraction of CD8^+^ T cells in both OC ascites and primary tumor expressed tissue-residency markers. A high number of these tissue-resident lymphocytes expressed NKG2A. NKG2A expression on CD8^+^ T cell has been reported as a late inhibitory receptor induced after repeated stimuli [51]. In colorectal cancer, the anti-tumor capacity of a subpopulation of NKG2A^+^ tumor infiltrating lymphocytes (TILs) is restored via blocking the NKG2A–HLA-E axis [11]. Moreover, the NKG2A blockade enhanced anti-tumor immunity in vivo in head and neck cancers [52] and the response of CD8^+^ T cells isolated from bladder tumors to HLA-E-expressing target cells [47].

The expression of HLA-E on OC tumors is heterogenous; however, in one study of 270 OC patients, the HLA-E expression level in the tumor was higher compared to that in the normal epithelium in 89% of the cases [53]. A high expression level of HLA-E in OC cell lines correlates with decreased CD8^+^ specific lysis in an NKG2A-dependent manner [54], and clinically, patients with high-level HLA-E-expressing tumors did not benefit from high numbers of infiltrating CD8^+^ cells, suggesting that high expression level of HLA-E neutralizes the effect of CD8^+^ cells due to the inhibition of their effector function [53]. The high NKG2A expression level in NK and CD8^+^ T cell subsets in the tumor microenvironment in OC, together with the high response rate in NKG2A^+^ traNK cells that we detected in this study, suggest that blocking the NKG2A – HLA-E interaction may be beneficial for patients with OC. Efforts are ongoing to unravel the potential use of NKG2A checkpoint inhibition to enhance the anti-tumor responses [18,19,55]. Recently, a phase III trial testing the NKG2A inhibitor, Monalizumab, in combination with the epidermal growth factor (EGFR) inhibitor, cetuximab, in patients with recurrent or metastatic squamous cell carcinoma of the head and neck was discontinued as it did not meet the pre-defined efficacy criteria (NCT04590963) [56]. However, several trials are ongoing evaluating the efficacy of Monalizumab, with one phase III trial on unresectable non-small cell lung cancer now recruiting (NCT05221840) [57].

Using a mouse OC model [48], we could demonstrate the infiltration of tissue-resident NK and T cells in the tumor microenvironment of the peritoneum upon tumor growth. Like in human patient samples, the mouse tumor-associated tissue-resident subsets expressed high levels of NKG2A. NKG2A expression correlated with better response towards the MOSEC ID8 cell line, where the increased expression of Qa-1^b^ diminished the response in NKG2A^+^ cells. These results further support that NKG2A expression may be a marker for anti-tumor immunity, and thus, a potential target for immunotherapeutic intervention in OC.

The origin of the NKG2A^+^ tissue-resident cell subsets detected in this study remains unknown. A recent report described the in vitro differentiation of ieILC1-like cells from conventional NK cells in response to IL-15 and TGF-β [38]. Another study reported the induced expression of CD103 on peripheral blood CD8^+^ T cells in a TGF-β-dependent and TCR-activating manner when they were co-cultured with either the OVCAR-3 cell line or OC tumor tissue [58]. Additionally, NKG2A expression is enhanced by in vitro IL-15 and/or TGF-β stimulation [19,47]. With increased IL-15 levels in OC ascites tumor microenvironment [59], we speculate that the cytokine milieu may be a driving factor of the differentiation from conventional NK cells to tissue-resident cells. Interestingly, high levels of CD103^+^ TILs are associated with a beneficial survival outcome for HGSC patients [58,60,61], suggesting that this population is an important immune compartment to consider when designing new OC therapies. Presumably, the best outcome of an NKG2A blockade in patients with OC may be obtained if it is used in combination with other immunotherapies that target T cells.

## 5. Conclusions

In conclusion, we report the presence of NK and CD8^+^ T cells with tissue-resident properties in both the metastatic ascites and the primary tumor microenvironment in HGSC. These tissue-resident cells are highly positive for the inhibitory receptor, NKG2A, and show responsiveness to OC cells. Together, this study suggests the presence of lymphocyte populations, which may be further evaluated as targets to improve immunotherapy in patients with OC.

## Data Availability

The data presented in this study are available on request from the corresponding authors.

## Supplementary Materials

The following supporting information can be downloaded at: https://www.mdpi.com/article/10.3390/cancers15133362/s1, Figure S1. Gating strategy to identify aNK cells. Figure S2. Phenotypic markers on aNK cells. Figure S3. (A) Gating strategy of one representative sample, gating for tissue-resident CD49^+^CD103^−^/CD49^+^CD103^+^/CD49^−^CD103^+^ or CD49a^−^CD103^−^ CD56^bright^ and CD56^dim^ NK cells from ascites of a patient with HGSC. (B) Expression of inhibitory receptors as noted within each subpopulation of ascites NK cells and patient-matched PBMCs. (C) NK subset distribution among ascites NK cells. Mean frequency of n = 5 samples; Figure S4. Expression of inhibitory receptors in HGSC-associated CD8^+^ T cells; Figure S5. Presence of tissue-resident CD4^+^ T cells in HGSC.

## Appendix A

**Table A1:** High-grade serous ovarian cancer patient characteristics, collection site and disease stage according to the FIGO (International Federation of Gynecology and Obstetrics) system.

Sample Type ^1^	Collection Site	Disease Stage	Age	Pharmacological Treatment ^2^	Other Diseases
PB; A	Sahlgrenska University Hospital	3C	42	No	No
PB; A	Sahlgrenska University Hospital	3C	73	No	No
PB; A	Sahlgrenska University Hospital	4	66	Norvasc, Levothyroxine	Hypertension, Hypothyroidism
PB; A	Sahlgrenska University Hospital	3A1	63	No	No
PB; A	Sahlgrenska University Hospital	4B	58	No	No
PB; T	Humanitas Research Hospital	2B	62	No	Hypothyroidism, Osteoporosis
PB; T	Humanitas Research Hospital	3C	75	Carboplatin, Paclitaxel, Gemcitabine, Bevacizumab	No
PB; T	Humanitas Research Hospital	3B	61	No	Hypertension
PB; T	Humanitas Research Hospital	4B	82	No	Arrythmia
PB; T	Humanitas Research Hospital	2C	58	Carboplatin, Paclitaxel, Niraparib	Previous TEP (pulmonary thromboembolism)
PB; A	Addenbrooke’s Hospital	3C	75	No	Restless legs syndrome, history of laminectomy, Meningioma, urge incontinence
PB; A	Addenbrooke’s Hospital	3C	70	Taxol, Carboplatin, Bevacizumab, Caelyx	Hysterectomy 30 years ago for fibroids
PB; A	Addenbrooke’s Hospital	3C	89	Taxol, Carboplatin	Oesophageal lichen planus, hearing loss

^1^ (PB = peripheral blood; A = ascites; T = primary tumor); ^2^ Upon sample collection.

**Table A2:** Antibodies used for mass cytometry analyses.

Reacts with	Antigen	Isotope	Clone	Source	Conjugated in-House	Used in tSNE Generation
Human	CD45	89Y	HI30	Fluidigm Sciences		yes
	CD3	Qdot605/170Er	UCHT1	Thermofisher		yes (not 2a)
	CD14	Qdot605/112Cd	Tük4	Thermofisher		yes (not 2a)
	CD19	Qdot605/112Cd	SJ25-C1	Thermofisher		yes (not 2a)
	HLA-DR	Qdot605/112Cd	Tü36	Thermofisher		yes (not 2a)
	CD57	115In	HCD57	BioLegend	yes	yes
	KIR2DS4	141Pr	FES172	Beckman Coulter, Brea, CA, USA	yes	yes
	CD103	142Nd	Ber-ACT8	BioLegend	yes	yes
	CD117	143Nd	104D2	Fluidigm Sciences		yes
	CD69	144Nd	FN50	Fluidigm Sciences		yes
	Granzyme B	146Nd	CLB-GB11	Novus, Singapore	yes	yes
	NKp30	148Nd	P30-15	BioLegend	yes	yes
	KIR2DL2/L3/S2	149Sm	GL183	Beckman Coulter	yes	yes
	CD107a	151Eu	H4A3	Fluidigm Sciences	yes	
	Eomes	152Sm	WD1928	eBioscience	yes	yes
	MIP1a	153Eu	1.2_3E8-2H6-2B6	Peprotech		
	CD96	154Sm	NK92.39	BioLegend	yes	yes
	CD56	155Gd	B159	Fluidigm Sciences		yes
	LILRB1	156Gd	GHI/75	Fluidigm Sciences		yes
	NKG2C	157Di	134591	R&D Systems, Minneapolis, MN, USA	yes	yes
	NKp44	160Gd	P44-8	BioLegend	yes	yes
	Tbet	161Dy	4B10	Fluidigm Sciences		yes
	NKp46	162Dy	BAB281	Fluidigm Sciences		yes
	CD49a	163Dy	TS2/7	Fluidigm Sciences		yes
	CD161	164Dy	HP-3G10	Fluidigm Sciences		yes
	CD127	165Ho	A019D5	Fluidigm Sciences		yes
	NKG2D	166Er	ON72	Fluidigm Sciences		yes
	KIR3DL1	167Er	DX9	Fluidigm Sciences		yes
	NKG2A	169Tm	Z199	Fluidigm Sciences		yes
	DNAM-1	171Yb	DX11	Fluidigm Sciences		yes
	Ki-67	172Yb	B56	Fluidigm Sciences		yes
	KIR2DL1	173Yb	143211	R&D Systems	yes	yes
	CD94	174Yb	HP-3D9	Fluidigm Sciences		yes
	AhR	175Lu	FF3399	eBioscience	yes	yes
	KIR2DL3	176Yb	180701	R&D Systems	yes	yes
	CD16	209Bi	3G8	Fluidigm Sciences		yes
	CD7	147Sm	CD7-6B7	Fluidigm Sciences		yes
	CD9	159Tb	SN4	Thermofisher	yes	yes
	EAT2	158Di			yes	yes
	GzmA	150Nd			yes	yes

**Table A3:** Antibodies used for flow cytometry analyses.

Reacts with	Antigen	Isotope	Clone	Source
Human	CD3	PerCp-Cy5.5	SK7	BD Biosciences
	CD3	BUV395	UCHT1	BD Horizon
	CD4	PerCP-Cy5.5	RPA-T4	Biolegend
	CD8a	FITC	RPA-T8	Biolegend
	CD14	PerCp-Cy5.5	M5E2	BD Biosciences
	CD19	PerCp-Cy5.5	HIB19	BD Biosciences
	CD16	BV786	3G8	BD Biosciences
	CD56	BV605	HCD56	Biolegend
	NKG2C	BUV737	134591	BD Biosciences
	NKG2A	PE-Cy7	Z199	Beckman Coulter
	KIR3DL1	FITC	DX9	BD Biosciences
	Pan-KIR2D	FITC	NKVFS1	Miltenyi Biotech
	HLA-E	PE	3D12	Biolegend
	CD103	R718	Ber-ACT8	BD Biosciences
	CD49a	APC	TS2/7	Biolegend
	CD94	BV480	HP-3D9	BD Biosciences
	CD107a	BUV395	H4A3	BD Biosciences
	INFg	BV650	4S.b3	Biolegend
	PD-1	PE-CF594	MIH4	BD Biosciences
	CTLA-4	BV421	BNI3	BD Biosciences
Mouse	CD45	AF700	30-F11	Biolegend
	CD45	BUV395	30-F11	BD Horizon
	CD3	BV785	17A2	Biolegend
	NK1.1	BUV395	PK136	BD Horizon
	NK1.1	PE-CF594	PK136	BD Pharmingen
	NKp46	BUV737	29A1.4	BD Pharmingen
	NKp46	PerCp-eFluor710	29A1.4	eBioscience
	NKG2A	PerCp-eFluor710	20d5	eBioscience
	NKG2A	APC	16A11	Biolegend
	CD49a	PE	Ha31/8	BD Pharmingen
	CD103	APC	2E7	Biolegend
	CD103	FITC	2E7	Biolegend
	PD-1	PE-Cy7	29F.1A12	Biolegend
	PD-1	APC	J43	eBioscience
	CTLA-4	BV605	UC0-4B9	Biolegend
	Ly49A	FITC	YE1/48.10.6	Biolegend
	EOMES	eFluor450	Dan11mag	eBioscience
	CD8a	BV605	53-6.7	Biolegend
	TIM-3	BV510	5D12/TIM-3	BD Biosciences
	LAG-3	PE-Cy7	C9B7W	Biolegend
	Granzyme B	Pacific Blue	GB11	Biolegend
	CD107a	PE	ID4B	Biolegend
