# Supplementary material for: Identification of Tissue-Resident Natural Killer and T Lymphocytes with Anti-Tumor Properties in Ascites of Ovarian Cancer Patients
Source: Cancers (Basel). 2023 Jun 27;15(13):3362. doi: 10.3390/cancers15133362 (PMC10340516; doi:10.3390/cancers15133362)
Supplement: Supplementary file 1 [file cancers-15-03362-s001.zip › cancers-2374008-supplementary.pdf]

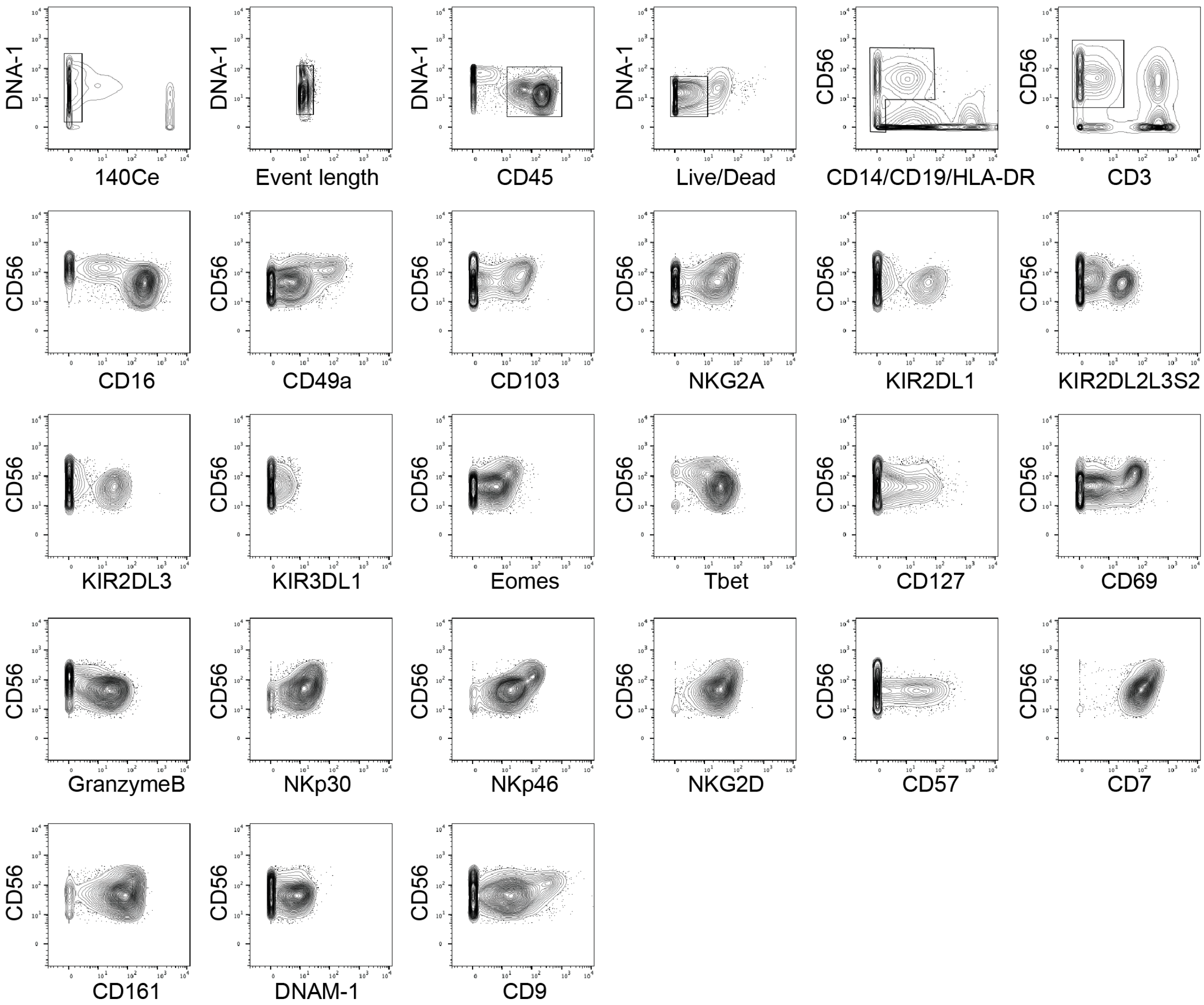

Supplementary Figure S1. Gating strategy to identify aNK cells. Representative gating strategy from one HGSC ascites samples are NK cells were defined as beads excluded, single cells, CD45^{+}, Live, CD14^{-}CD19^{-}HLA-DR^{-}, CD3^{-} CD56^{+}. Cells are further divided into tissue-resident cells based on their expression of CD49a and CD103, or CD56^{bright} and CD56^{dim} pb-like cells, and their expression of specified phenotypic markers evaluated.

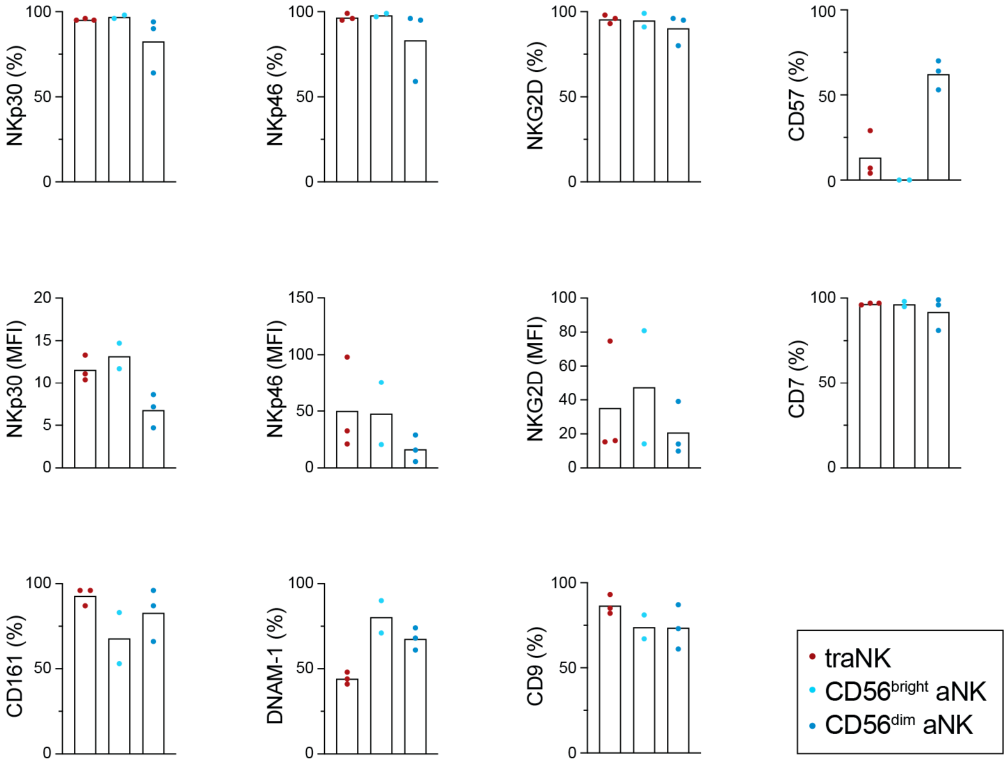

Supplementary Figure S2. Phenotypic markers on aNK cells. Expression of phenotypic markers as specified on tissue-resident (traNK) and CD56^{bright} and CD56^{dim} aNK cells within the OC ascites compartment. n=3.

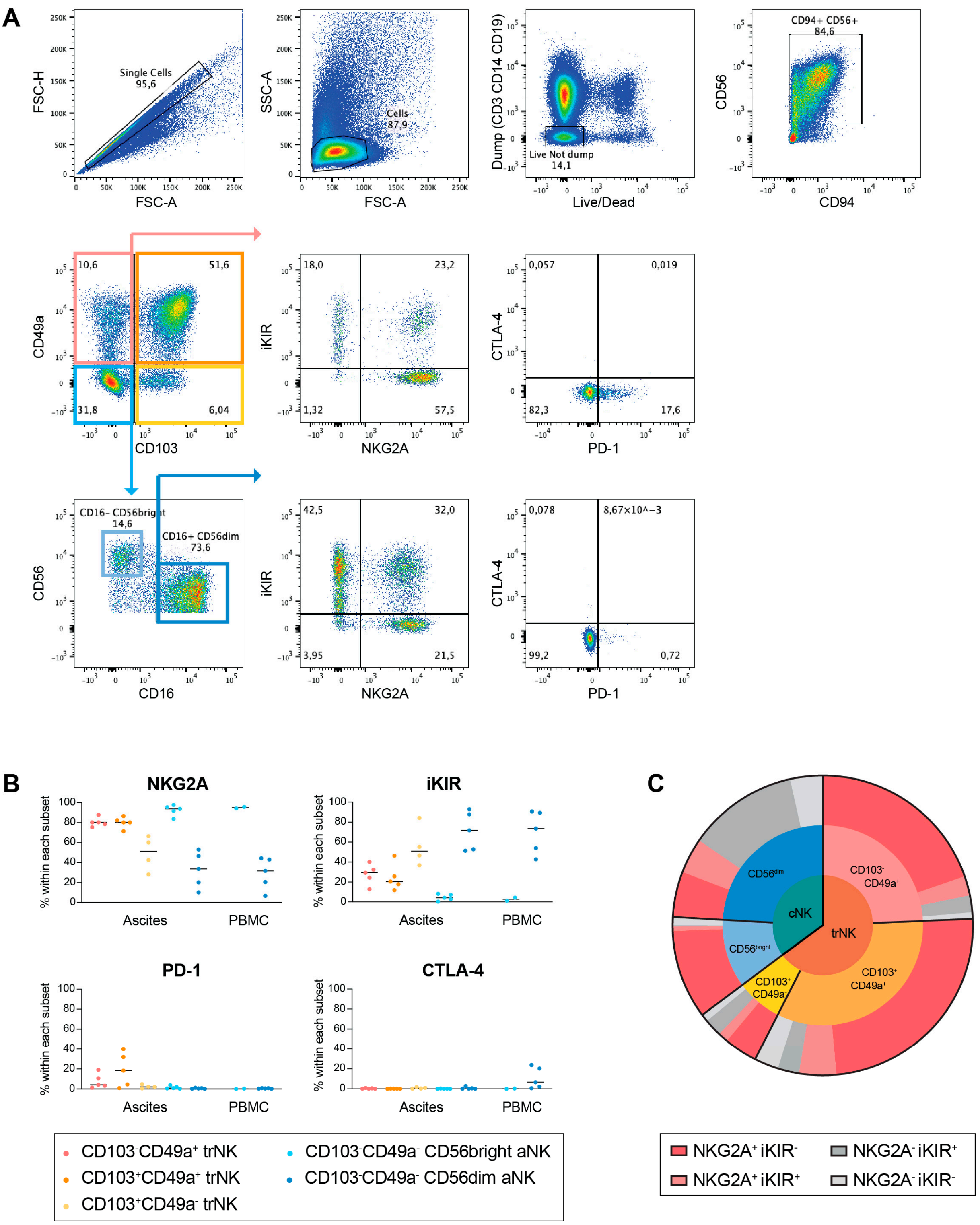

Supplementary Figure S3. A) Gating strategy of one representative sample, gating for tissue-resident CD49^{+}CD103^{-}/CD49^{+}CD103^{+}/CD49^{-}CD103^{+} or CD49a^{-}CD103^{-} CD56^{bright} and CD56^{dim} NK cells from ascites of a patient with HGSC. B) Expression of inhibitory receptors as noted within each subpopulation of ascites NK cells and patient-matched PBMCs. C) NK subset distribution among ascites NK cells. Mean frequency of n=5 samples.

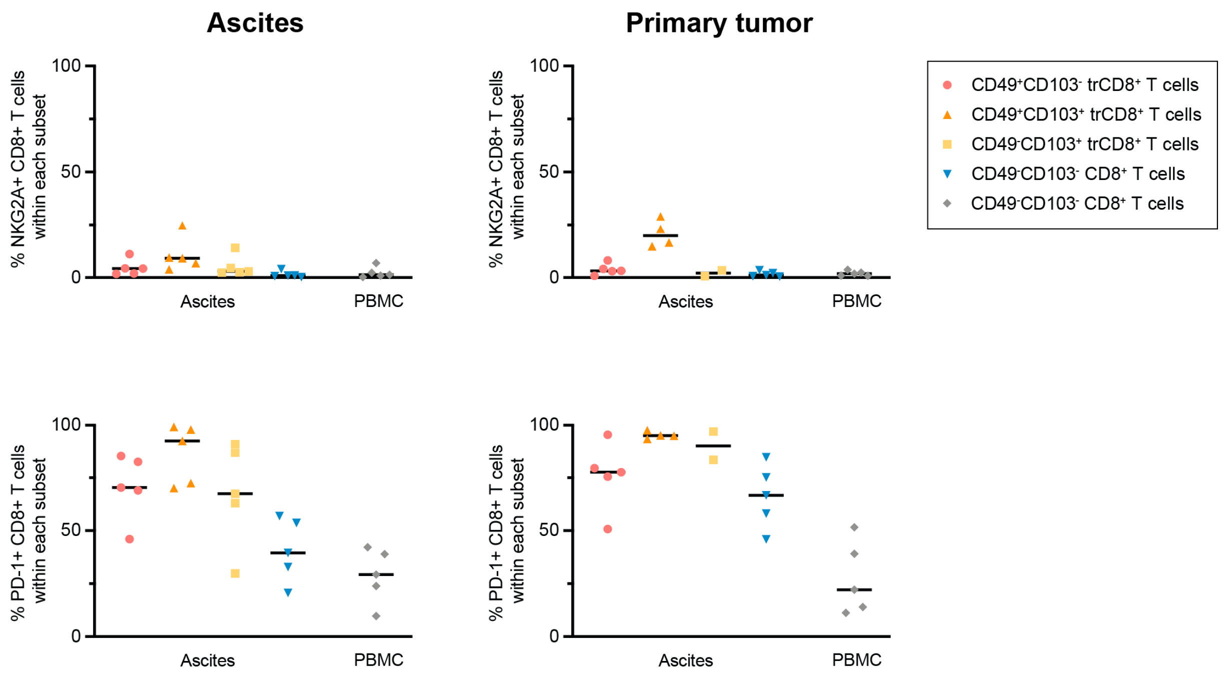

Supplementary Figure S4. **Expression of inhibitory receptors in HGSC-associated CD8⁺ T cells**. Frequency of NKG2A and PD-1 expression on tissue-resident and CD8⁺ T cell subsets in ascites and primary tumor, with patient-matched PBMC for each site. n=5 (ascites), n=5 (primary tumor).

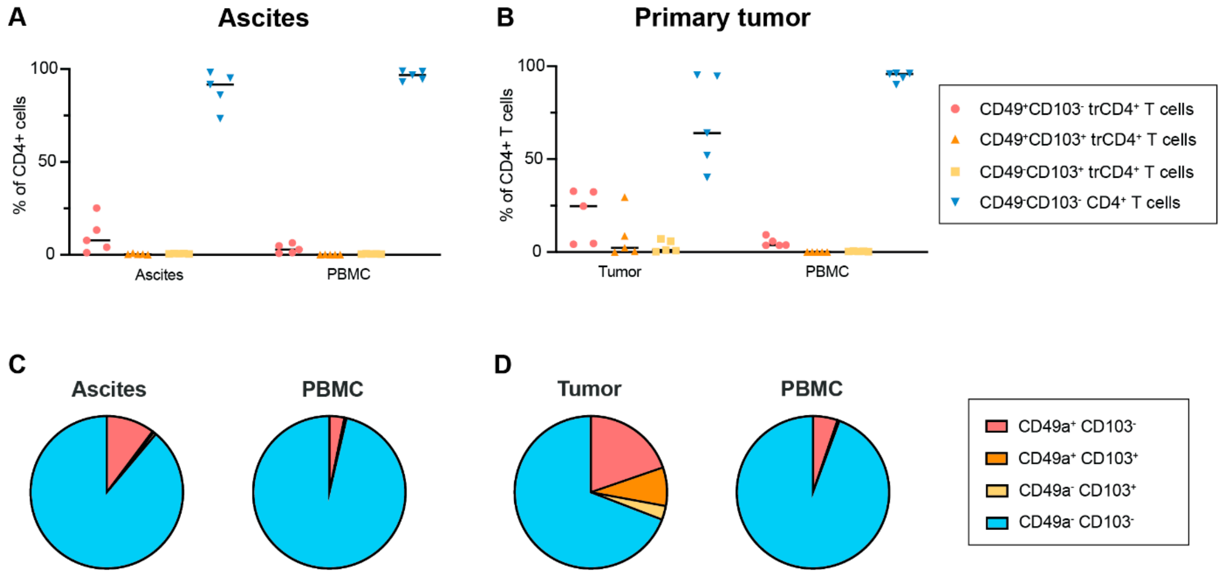

Supplementary Figure S5. **Presence of tissue-resident CD4⁺ T cells in HGSC.** A,B) Distribution of tissue-resident (tr) and pb-like CD4⁺ T cells in ascites (A) and primary tumor (B), with patient-matched PBMC for each site. C,D) Mean distribution of data in A and B. n=5 (ascites), n=5 (primary tumor).
